# Rosiglitazone Elicits an Adiponectin-Mediated Insulin-Sensitizing Action at the Adipose Tissue-Liver Axis in Otsuka Long-Evans Tokushima Fatty Rats

**DOI:** 10.1155/2018/4627842

**Published:** 2018-08-27

**Authors:** Jia Li, Yao-Ming Xue, Bo Zhu, Yong-Hua Pan, Yan Zhang, Chunxia Wang, Yuhao Li

**Affiliations:** ^1^Department of Endocrinology, General Hospital of Guangzhou Military Command of PLA, Guangzhou 510010, China; ^2^Department of Endocrinology & Metabolism, Nanfang Hospital, Southern Medical University, Guangzhou 510515, China; ^3^Department of Pharmacy, Nanfang Hospital, Southern Medical University, Guangzhou 510515, China; ^4^Guangdong Key Laboratory of New Drug Screening, School of Pharmaceutical Sciences, Southern Medical University, Guangzhou 510515, China; ^5^Endocrinology and Metabolism Group, Sydney Institute of Health Sciences/Sydney Institute of Traditional Chinese Medicine, Sydney, NSW 2000, Australia

## Abstract

Rosiglitazone is an agonist of peroxisome proliferator-activated receptor- (PPAR-) *γ* that is principally associated with insulin action. The exact mechanisms underlying its insulin-sensitizing action are still not fully elucidated. It is well known that adiponectin mostly secreted in adipose tissue is an insulin sensitizer. Here, we found that treatment of Otsuka Long-Evans Tokushima Fatty (OLETF) rats with rosiglitazone (3 mg/kg, once daily, by oral gavage for 33 weeks) attenuated the increase in fasting plasma insulin concentrations and the index of the homeostasis model assessment of insulin resistance along with the age growth and glucose concentrations during an oral glucose tolerance test. In addition, the increase in plasma alanine aminotransferase activity, concentrations of fasting plasma nonesterified fatty acids and triglyceride, and hepatic triglyceride content was also suppressed. The hepatic protein expression profile revealed that rosiglitazone increased the downregulated total protein expression of insulin receptor substrate 1 (IRS-1) and IRS-2. Furthermore, the treatment suppressed the upregulated phosphorylation of IRS-1 at Ser^307^ and IRS-2 at Ser^731^. The results indicate that rosiglitazone ameliorates hepatic and systemic insulin resistance, hepatic inflammation, and fatty liver. Mechanistically, rosiglitazone suppressed hepatic protein overexpression of both phosphorylated nuclear factor- (NF-) *κ*Bp65 and inhibitory-*κ*B kinase-*α*/*β*, a transcription factor that primarily regulates chronic inflammatory responses and the upstream NF-*κ*B signal transduction cascades which are necessary for activating NF-*κ*B, respectively. More importantly, rosiglitazone attenuated the decreases in adipose adiponectin mRNA level, plasma adiponectin concentrations, and hepatic protein expression of adiponectin receptor-1 and receptor-2. Thus, we can draw the conclusion that rosiglitazone elicits an adiponectin-mediated insulin-sensitizing action at the adipose tissue-liver axis in obese rats. Our findings may provide new insights into the mechanisms of action of rosiglitazone.

## 1. Introduction

Insulin resistance is the cord that runs through various modern chronic illnesses, of which obesity, dyslipidemia, hypertension, type 2 diabetes, nonalcoholic fatty liver diseases, and cardiovascular disease are included [1]. The liver, adipose tissue, and skeletal muscle are the main insulin-targeted organs. Thus, the insulin resistance in the liver, skeletal muscle, and adipose tissue plays an important role in systemic insulin resistance.

The thiazolidinediones (TZDs) are agonists of peroxisome proliferator-activated receptor- (PPAR-) *γ*, a nuclear transcription factor principally associated with insulin action, lipid and glucose metabolism, and energy homeostasis [2, 3]. Large amounts of studies have shown that TZDs improve insulin sensitivity in various animal models with insulin resistance and patients with type 2 diabetes [2]. Rosiglitazone is one of the currently used TZDs/PPAR-*γ* agonists in the treatment of type 2 diabetes. It has been demonstrated that rosiglitazone improves insulin resistance in most tissues, such as adipose tissue [4] and tissues of the skeletal muscle [5], liver [6], myocardium [7], blood vessel [8], and kidney [9]. There is, however, still an important but unresolved question associated with the direct targets and action mechanisms of the TZD.

It has been suggested that improvement of peripheral insulin action by TZDs is possibly mediated by secondary mechanisms [2]. Adipose tissue is an endocrine organ that plays a substantial role in metabolism. The adipose tissue-liver axis has been suggested to mediate many systemic or local pathological changes and the effects of pharmacological interventions. The subcutaneous adipose tissue-liver axis has been found to control hepatic gluconeogenesis [10]. The adipose tissue-liver axis is responsible for chronic alcohol exposure-induced disturbance in lipid homeostasis in mice [11] and plays an important role in both initiation and progression of chronic alcohol consumption-induced liver damage [12]. The axis mediates rosiglitazone-elicited improvement of ethanol-induced abnormalities of lipid metabolism in mice [13], *α*-linolenic acid-rich-flaxseed oil-elicited amelioration of alcoholic fatty liver in mice [14], and scopoletin-provoked improvement of alcohol-induced lipid dysregulation and inflammation in rats [15]. Adiponectin is mostly produced in adipose tissue [16]. The communication between the adipose tissue and other tissues may play an important role in adiponectin-elicited amelioration of insulin resistance [17]. Generally, hepatic steatosis and severity of nonalcoholic fatty liver diseases may be predicted by adiponectin level, and the latter is also closely associated with regulation of hepatic insulin signaling [16]. Two distinct adiponectin receptors have tissue-specific distributions: adiponectin receptor 2 (ADNR-2) is more abundant in the liver, and ADNR-1 in skeletal muscles [16]. It is known that in the liver, adiponectin exerts its insulin-sensitizing action via its receptors; the underlying mechanisms are associated with suppression of inflammation through the nuclear factor- (NF-) *κ*B pathway and inhibition of gluconeogenesis, de novo lipogenesis, and free fatty acid influx and enhancement of fatty acid *β*-oxidation through the 5-AMP-activated protein kinase (AMPK) and PPAR-*α* pathways [16].

PPAR-*γ* is predominantly expressed in adipose tissue, whereas it presents much less in other tissues, such as only about 10% of the level of adipose tissue expression in the skeletal muscle [2, 18]. Rosiglitazone treatment has been found to improve hypoadiponectinemia in patients with nonalcoholic steatohepatitis [19]. Rosiglitazone also attenuated high-fat diet-induced downregulation of mRNA and protein expression of ADNR-1 and ADNR-2 in visceral fat and hypoadiponectinemia in rats [20]. It appears that adiponectin plays an important role in the TZD-elicited insulin sensitization [16]. However, the exact action mechanisms of the TZD, especially the role mediated by adiponectin at the adipose tissue-liver axis in obesity, remain unclear.

Otsuka Long-Evans Tokushima Fatty (OLETF) rat is a spontaneously diabetic model with obesity, insulin resistance, and hyperlipidemia; the clinical and pathological features of OLETF rats resemble those of human type 2 diabetes [5, 21, 22]. In the present study, we investigated whether rosiglitazone-elicited insulin sensitization is mediated by adipose tissue-derived adiponectin at the adipose tissue-liver axis in the genetically occurred rat model.

## 2. Materials and Methods

### 2.1. Animals, Chemical, and Diet

Male OLETF rats aged 4 weeks and their age-matched lean nondiabetic counterparts, Long-Evans Tokushima Otsuka (LETO) rats, were provided by Otsuka Pharmaceutical Co. (Tokushima, Japan). The regular standard diet (61% carbohydrate, 22% protein, and 3% crude fat) was supplied by the Experimental Animal Center, Southern Medical University, Guangzhou, China. Rosiglitazone 5-[{4-[2-(methyl-2-pyridinylamino)ethoxy]-phenyl}methyl]-2,4-thiazolidinedione was purchased from GlaxoSmithKline Pharmaceuticals (Philadelphia, PA, USA).

#### 2.1.1. Experimental Protocol

All animal procedures were conducted following international, national, and institutional rules regarding animal experimentation and approved by the Animal Ethics Committee, Southern Medical University, Guangzhou, China. Rats were housed in the specific pathogen-free animal room (a 12 h light/dark cycle, 21 ± 1°C, 55 ± 5% relative humidity) in the Experimental Animal Center of Southern Medical University. Animals were allowed free access to water and the standard diet.

Sixteen OLETF rats at the age of 8 weeks were divided randomly into a OLETF control group (OLETF-C) and rosiglitazone-treated group (OLETF-RSG) (*n* = 8 each), while eight LETO rats were treated as a normal control group (LETO-C). To avoid stress and accurately calculate chow intake, only 2 animals were housed in a cage. The OLETF-RSG group was administered rosiglitazone (3 mg/kg, dissolved in 5 mL distilled water, by a gavage method, at 10:00 am, once daily) until the age of 40 weeks. OLETF-C and LETO-C groups were administered distilled water only. The body weight and consumed diet (per cage) were measured weekly. At 8, 32, and 40 weeks of age, all rats were deprived of diet but still had free access to water overnight, and blood samples were collected under anesthesia with pentobarbital sodium for determination of plasma glucose, insulin, and adiponectin concentrations. At the end of 40 weeks, all rats were sacrificed by prompt dislocation of the neck vertebra under anesthesia. Liver and visceral (epididymal, perirenal, and omental) fats were collected and weighed. The ratio of visceral fat to body weight was calculated accordingly. Segments of the liver and fat were flash frozen in liquid nitrogen and stored at −80°C for subsequent determination of triglyceride content and/or gene/protein expression. A portion of the liver was fixed with 10% formalin and embedded in paraffin for histological examination.

### 2.2. Oral Glucose Tolerance Test (OGTT)

OGTT was conducted at the age of 40 weeks. After overnight fasting, blood was sampled using a tail-cutting method, and glucose concentration was detected with a OneTouch monitoring system (LifeScan, CA, USA). A glucose solution (2 g/kg in 5 mL) was given orally. Blood glucose concentrations were determined again at 30, 60, 90, and 120 min after glucose feeding. The areas under the curve (AUC) of blood glucose concentrations during OGTT and the index of the homeostasis model assessment of insulin resistance (HOMA-IR) {[fasted insulin (*μ*IU/mL) × fasted glucose (mM)]/22.5} [23] were calculated, respectively. In addition, plasma triglyceride (kits from Nanjing Jiancheng Bioengineering Institute, Nanjing, China) and nonesterified fatty acid (NEFA) (NEFA-C kit, Wako, Osaka, Japan) concentrations and alanine aminotransferase (ALT) activity (kit from Kexin Institute of Biotechnology, Shanghai, China) before glucose feeding were also determined using enzymatic methods, respectively.

### 2.3. Histological Examinations

Three-micron-thick sections of the liver samples embedded in paraffin were stained with standard hematoxylin-eosin to observe the histological changes (IX-81, Olympus Corporation, Tokyo, Japan).

### 2.4. Determination of Liver Triglyceride Content

Hepatic triglyceride content was determined as described previously [24]. Briefly, the homogenate of the liver (100 mg tissue/2 mL) was centrifuged (3000 rpm), and supernatant triglyceride concentration was determined enzymatically (kits from Nanjing Jiancheng Bioengineering Institute, Nanjing, China).

### 2.5. Real-Time PCR

Total RNA was isolated (TRIzol method) from adipose tissue of individual rats, and cDNA was synthesized (M-MLV RTase cDNA Synthesis) according to the manufacturer's instructions (kit from Takara, Dalian, China). Real-time PCR was performed with the LIGHTCYCLER 480 Real-Time PCR Detection System (LIGHTCYCLER 480, Roche, Germany) using the SYBR® Premix Ex Taq™ II (Takara, Dalian, China). The sequences of primers are as follows: adiponectin (forward: 5′TCACTCAGCATTCAGCGTAG3′ and reverse: 5′CTGATACTGGTCGTAGGTGAAG3′) and GAPDH (forward: 5′CCTTCATTGACCTCAACTACATGG3′ and reverse: 5′GCAGTGATGGCATGGACTGTGGT3′). Each adiponectin mRNA expression was standardized against GAPDH. The result in the LETO group was arbitrarily assigned as 1.

### 2.6. Western Blot Analysis

Briefly, proteins were extracted from each frozen liver tissue. Thirty micrograms of protein was fractionated on 10% SDS-polyacrylamide gels and transferred to polyvinylidene fluoride membrane. The membranes were blocked with 5% nonfat milk for 1 h at room temperature and incubated at 4°C overnight with primary antibodies specific for total insulin receptor substrate-1 (tIRS-1) and tIRS-2 (dilution 1 : 500, Cell Signaling Technologies, Beverly, MA, USA), phosphorylated IRS-1 at Ser^307^ (pSer^307^) (dilution 1 : 300, Cell Signaling Technologies, Beverly, MA, USA), phosphorylated IRS-2 at Ser^731^ (pSer^731^) (dilution 1 : 200, Abcam plc, Cambridge, UK), phosphorylated inhibitory-*κ*B kinase-*α*/*β* (IKK-*α*/*β*) (dilution 1 : 1000, Cell Signaling Technologies, Beverly, MA, USA), and ADNR-1 and ADNR-2 (dilution 1 : 1000, Bioss Co. Ltd., Beijing, China), followed by anti-rabbit horseradish peroxidase-conjugated IgG (Santa Cruz Biotechnology, Santa Cruz, CA, USA) as the second antibody. A polyclonal rabbit *β*-actin antibody (Cell Signaling Technologies, Beverly, MA, USA) was used as the loading control to normalize the signal obtained for proteins. The bound antibody was visualized with an enhanced chemiluminescence system (ECL, Amersham, IL, USA) and X-ray film (Fujifilm, Uetake, Japan). The results were analyzed by Gel Pro Analyzer software (Media Cybernetics Inc., USA) to measure the hue values of the target proteins in each group. Levels in the LETO group were arbitrarily assigned a value of 1.

### 2.7. Immunohistochemistry

4 *μ*m thick serial sections of the paraffin-prepared hepatic tissues were dewaxed, rehydrated in multiple-graded ethanol solutions, and treated with 3% hydrogen peroxide to inactivate endogenous peroxidases. The sections were incubated with a primary mouse monoclonal phosphorylated NF-*κ*Bp65 (pNF-*κ*Bp65) antibody (dilution of 1 : 100) (Santa Cruz Biotechnology, Santa Cruz, CA, USA) at 4°C overnight and then with a biotinylated secondary antibody at room temperature. The immune reaction was revealed with diaminobenzidine (DAB-DAKO, Carpinteria, CA, USA) and hydrogen peroxide. The preparations were lightly counterstained with hematoxylin, mounted with Permount, and examined by a light microscope (OLYMPUS-BX51-DP70, Olympus, Japan) connected to an interactive image analysis system (Image-Pro Plus, Media Cybernetics, USA). Ten visual fields each slide were randomly selected and measured with Image-Pro Plus image analysis software. The positive unit was calculated according to the following formula and used to evaluate the expression level of pNF-*κ*Bp65: (G*α* − G*β*)/area, where G*α* is the mean hue value of pNF-*κ*Bp65 positive expression, G*β* is the mean hue value of the background, and area is the percentage of the pNF-*κ*Bp65 positive area under the full microscopic visual field [25].

### 2.8. Statistical Analysis

The quantitative data were expressed as mean ± SD. The statistical analysis was performed using the SPSS software program (version 13.0; SPSS Inc., Chicago, USA). The comparison of quantitative data between 2 samples was performed with *t*-test of 2 independent samples. The single-factor ANOVA test was adopted for multiple comparisons. For comparison of any 2 groups, the Student–Newman–Keuls method was adopted in the cases of homoscedasticity; otherwise, Tamhane's T2 method was used. For the bivariate correlation tests between factors, the nonparameter methods were adopted with a rank correlation coefficient (Spearman's rank correlation coefficient). Differences with *P* < 0.05 were considered to be statistically significant.

## 3. Results

### 3.1. Effects on Plasma Concentrations of Glucose, Insulin, the HOMA-IR Index, Triglyceride, and NEFA in Rats

**Figure 1:**
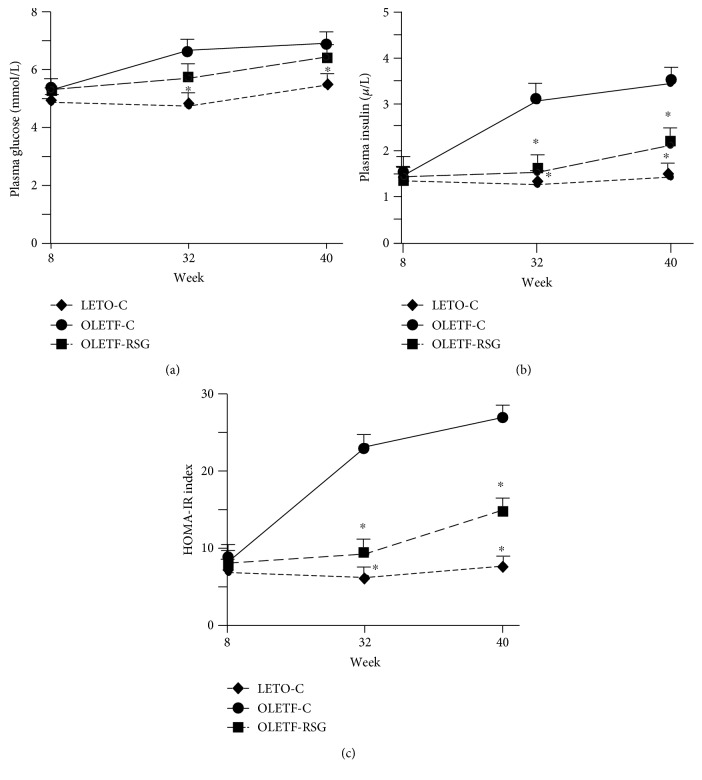
Fasting plasma glucose (a) and insulin (b) concentrations and the HOMA-IR index (c) at the ages of 8 weeks, 32 weeks, and 40 weeks in LETO-C, OLETF-C, and OLETF-RSG groups. Data are means ± SD (*n* = 8 each group) versus OLETF group (^∗^*P* < 0.05).

Under fasting condition, there was no significant difference in plasma glucose and insulin concentrations and the HOMA-IR index among the LETO-C, OLETF-C, and OLETF-RSG groups before the treatment (week 8). Along with the age increase, plasma glucose concentrations in the OLETF-C group were slightly but significantly higher than those in the LETO-C group at weeks 32 and 40 (Figure 1(a)). Strikingly, plasma insulin concentrations (Figure 1(b)) and the HOMA-IR index (Figure 1(c)) were much higher in the OLETF-C group than in the LETO-C group. Treatment with rosiglitazone did not significantly affect the glucose concentrations, whereas it significantly suppressed the increase in plasma insulin concentrations and the HOMA-IR index at the time points (Figures 1(a)–1(c)).

**Figure 2:**
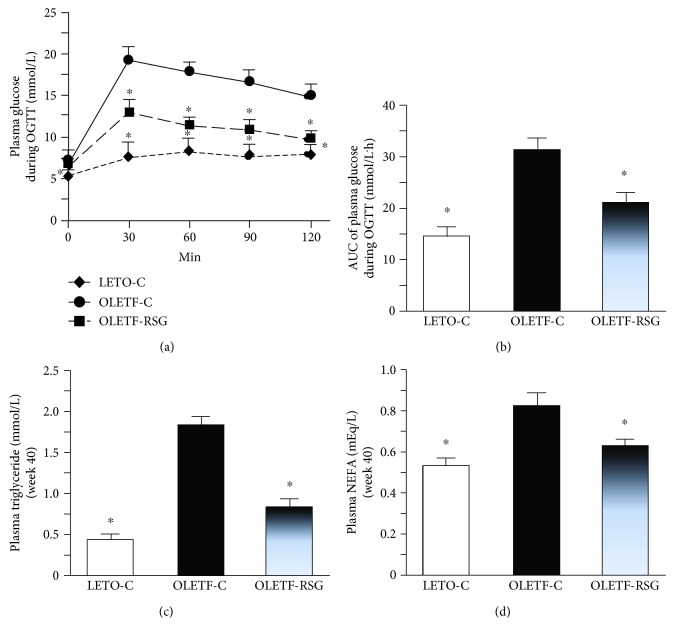
Blood glucose concentrations at different time points (a), the AUC of glucose concentrations (b) during oral glucose tolerance test (OGTT), and fasting plasma triglyceride (c) and NEFA (d) concentrations in LETO-C, OLETF-C, and OLETF-RSG groups. Data are means ± SD (*n* = 8 each group) versus OLETF group (^∗^*P* < 0.05).

During OGTT at week 40, blood glucose concentrations in the OLETF-C group were increased more than those in the LETO-C group (Figure 2(a)) after glucose feeding. Subsequently, the AUC of blood glucose concentrations was also higher (Figure 2(b)). In addition, OLETF-C rats also showed higher plasma triglyceride (Figure 2(c)) and NEFA (Figure 2(d)) concentrations than the LETO-C group. Rosiglitazone treatment attenuated the increases of these parameters (Figures 2(a)–2(d)).

### 3.2. Effects on Hepatic Protein Expression of tIRS-1, tIRS-2, Phosphorylated IRS-1 at pSer^307^, and IRS-2 at Ser^731^ in Rats

**Figure 3:**
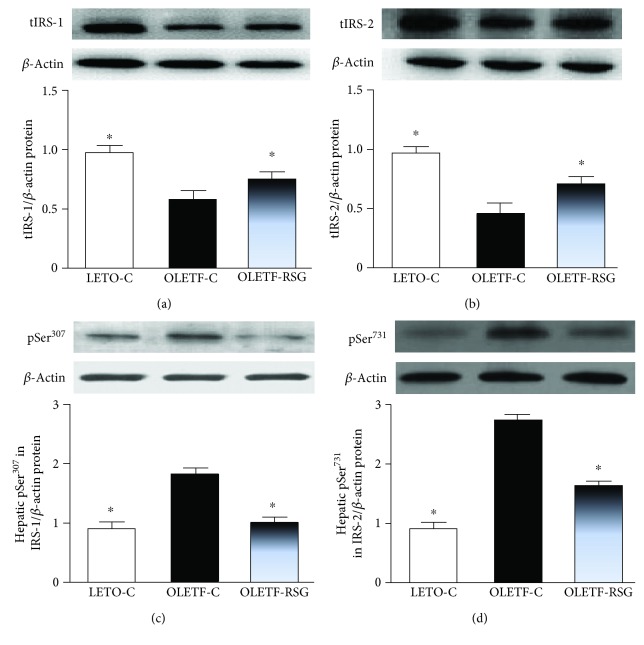
Hepatic protein expression of total IRS-1 (tIRS-1) (a), total IRS-2 (tIRS-2) (b), pSer^307^ in IRS-1 (c), and pSer^731^ in IRS-2 (d) in LETO-C, OLETF-C, and OLETF-RSG groups. Protein expression was determined by Western blot. The result in the LETO-C group was arbitrarily assigned a value of 1. Data are means ± SD (*n* = 8 each group) versus OLETF group (^∗^*P* < 0.05).

By Western blot, the OLETF-C group showed decreases in tIRS-1 (Figure 3(a)) and tIRS-2 (Figure 3(b)) protein expression in the liver. More importantly, both pSer^307^ in IRS-1 (Figure 3(c)) and Ser^731^ in pIRS-2 (Figure 3(d)) were increased, compared to those in the LETO-C group. Treatment with rosiglitazone upregulated the decreased expression of tIRS-1 and tIRS-2 and suppressed the increased serine phosphorylation of IRS-1 and IRS-2 proteins (Figures 3(a)–3(d)).

### 3.3. Effects on Hepatic Lipid Accumulation and Inflammatory Parameters in Rats

**Figure 4:**
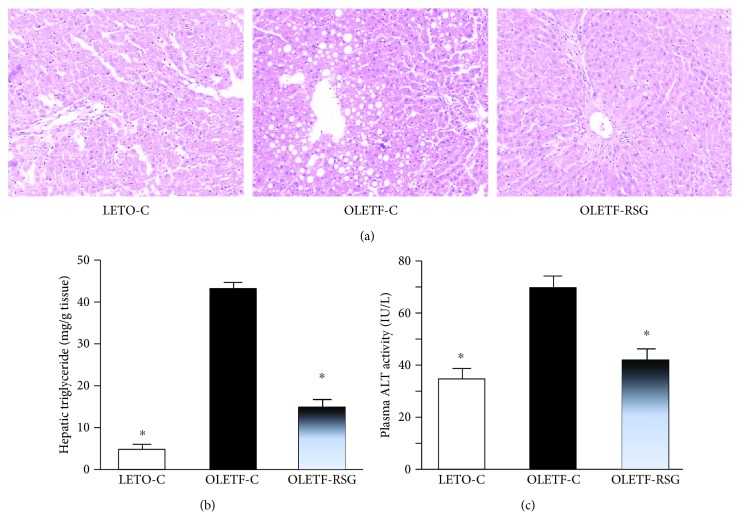
Representative images showing histology of the liver (hematoxylin and eosin staining) (a, ×50), hepatic triglyceride content (b), and fasting plasma alanine aminotransferase (ALT) (c) activity in LETO-C, OLETF-C, and OLETF-RSG groups. Data are means ± SD (*n* = 8 each group) versus OLETF group (^∗^*P* < 0.05).

Pathological examination revealed that OLETF rats at the age of 40 weeks had plenty of vacuolar changes in hepatocytes (Figure 4(b)), whereas no vacuolar change was found in LETO rats (Figure 4(a)). Furthermore, hepatic triglyceride accumulation in OLETF rats was 8-fold of that in LETO rats (Figure 4(b)). The rosiglitazone-treated group showed only slight vacuolarized hepatocytes (Figure 4(a)) and much lower hepatic triglyceride content (Figure 4(b)).

**Figure 5:**
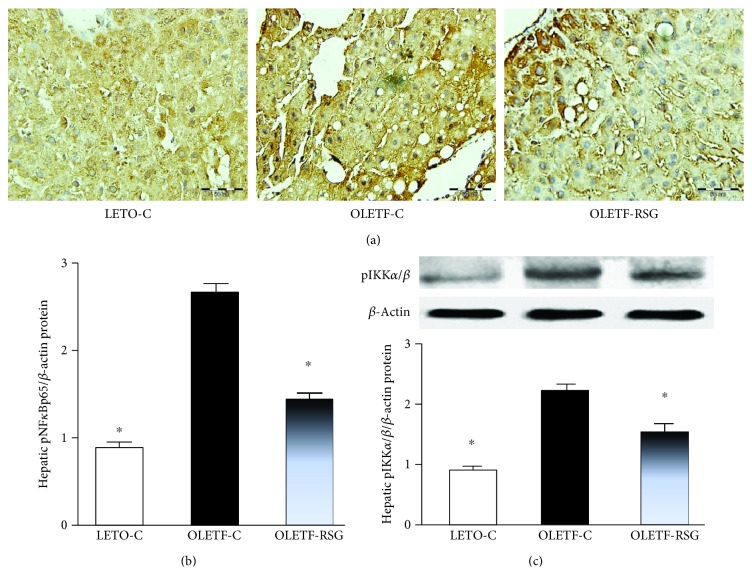
Representative images showing immunohistochemical staining (a, ×200) and expression level (b) of hepatic phosphorylated NF-*κ*Bp65 (pNF-*κ*Bp65) protein and hepatic pIKK-*α*/*β* protein expression by Western blot (c) in LETO-C, OLETF-C, and OLETF-RSG groups. The result in the LETO-C group was arbitrarily assigned a value of 1. Data are means ± SD (*n* = 8 each group) versus OLETF group (^∗^*P* < 0.05).

Plasma ALT activity in OLETF rats was increased, compared to that in LETO rats (Figure 4(c)), indicating liver lesions. Furthermore, hepatic expression of pNF-*κ*Bp65 (Figures 5(a) and 5(b)) and pIKK-*α*/*β* (Figure 5(c)) proteins in OLETF rats was upregulated. Rosiglitazone treatment suppressed the increased plasma ALT activity and upregulation of hepatic expression of the proteins in OLETF rats (Figures 4(c) and 5(a)–5(c)).

### 3.4. Effects on Food Intake, Body Weight, the Ratio of Visceral Fat to Body Weight, Adipose Adiponectin Expression, Plasma Adiponectin Concentration, and Hepatic Protein Expression of ADNRs-1/2 in Rats

**Figure 6:**
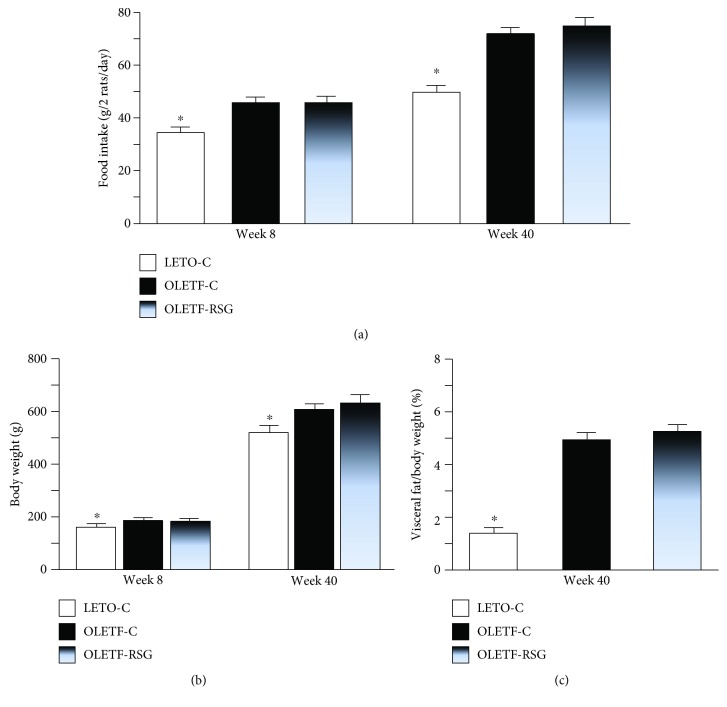
Intakes of laboratory chow (a), body weight prior to and after treatment (b), and the ratio of visceral fat to body weight (c) in LETO-C, OLETF-C, and OLETF-RSG groups. Data are means ± SD (*n* = 8 each group) versus OLETF group (^∗^*P* < 0.05).

OLETF rats ate more than LETO rats (Figure 6(a)) and were heavier (Figure 6(b)) since 8 weeks of age. The ratio of visceral fat to body weight at week 40 was increased to over 3-fold of that in LETO rats (Figure 6(c)). Treatment with rosiglitazone did not affect food intake, body weight, and the ratio (Figures 6(a)–6(c)).

**Figure 7:**
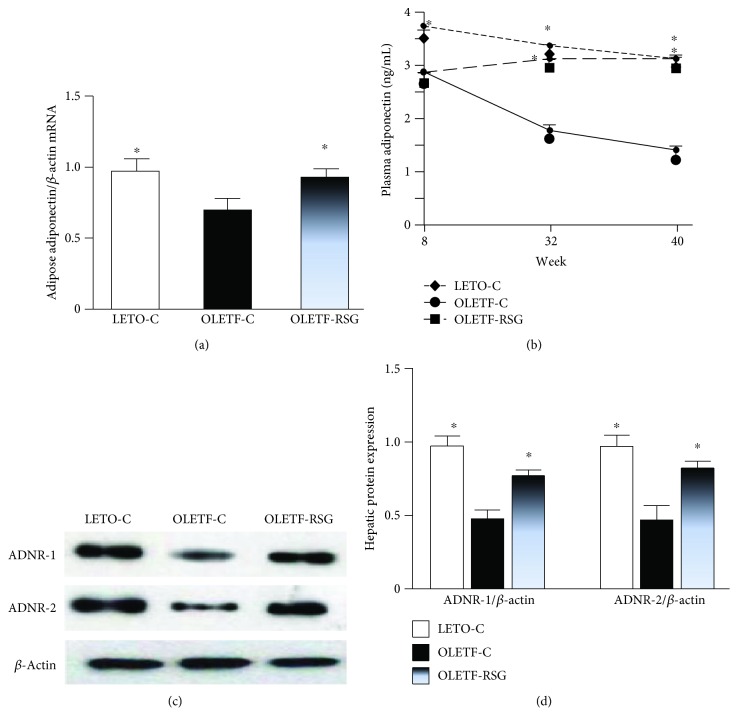
Adipose mRNA expression of adiponectin (a), plasma adiponectin concentrations (b), and hepatic protein expression of ADNR-1 and ADNR-2 in LETO-C, OLETF-C, and OLETF-RSG groups. Protein expression was determined by Western blot. The result in the LETO-C group was arbitrarily assigned a value of 1. Data are means ± SD (*n* = 8 each group) versus OLETF group (^∗^*P* < 0.05).

In contrast to the increased body weight and the ratio of visceral fat to body weight, real-time PCR analysis revealed that adipose adiponectin mRNA expression in the OLETF-C group was downregulated, compared to that in the LETO-C group (Figure 7(a)). Moreover, plasma adiponectin concentrations were decreased since the age of 8 to 40 weeks (Figure 7(b)). Rosiglitazone treatment restored downregulation of adipose adiponectin expression and decreased plasma adiponectin concentration in OLETF rats (Figures 7(a) and 7(b)).

By Western blot, hepatic expression of ADNR-1 and ADNR-2 (Figures 7(c) and 7(d)) proteins in OLETF rats was markedly downregulated to half of that in LETO rats. Rosiglitazone treatment restored downregulation of protein expression of the receptors near normal levels (Figures 7(c) and 7(d)).

## 4. Discussion

Insulin resistance is closely associated with a wide array of other disorders, such as hyperlipidemia, and also a fundamental aspect of the etiology of type 2 diabetes [26]. Obesity is linked to and engenders insulin resistance; the enlarged adipose tissue mass causes systemic insulin resistance/hyperinsulinemia [26]. OLETF rat is a spontaneously diabetic model with obesity and polyphagia and develops a late onset of hyperglycemia [21]. The present study clearly demonstrated that intervention of OLETF rats with rosiglitazone attenuated hyperinsulinemia, glucose intolerance, and hyperlipidemia. However, rosiglitazone did not affect the mild hyperglycemia, food intake, body weight, and the ratio of visceral fat to body weight. Thus, these results suggest that rosiglitazone improves insulin sensitivity not directly by interfering with obesity but possibly by interacting on obesity-associated downstream factors.

The liver is responsible for endogenous glucose output to circulation; thus, the liver plays a central role in lipid and glucose metabolism and contributes to whole-body glucose homeostasis [27]. The HOMA-IR index has been suggested to evaluate hepatic insulin sensitivity [23]. The insulin receptor after being activated by binding with insulin promotes the tyrosine phosphorylation of a number of cellular proteins including the IRS proteins which are major physiological targets of the activated insulin receptor kinase. Hepatic IRS-1 and IRS-2 play a pivotal role in mediating insulin-dependent regulation of glucose and lipid metabolism; dysregulation of abundance and/or phosphorylation status of IRS-1 and IRS-2 in the liver are significant factors in the pathogenesis of insulin resistance and type 2 diabetes [28]. Generally speaking, serine phosphorylation is harmful to IRS signaling. IRS serine phosphorylation is a physiological feedback mechanism in insulin signaling that is hijacked by metabolic and inflammatory stresses to promote insulin resistance. Thus, the upregulated serine phosphorylation of IRS-1 plays a key role in the pathogenesis of insulin resistance [29]. Serine phosphorylation of IRS-1 suppresses insulin signal transduction in a variety of cell backgrounds, which might contribute to peripheral insulin resistance [30]. IRS-2 is a major player of hepatic insulin action [31]. In the present study, treatment with rosiglitazone inhibited the increase in the HOMA-IR index in OLETF rats, indicating amelioration of hepatic insulin resistance. The upregulation of the decreased hepatic protein expression of tIRS-1 and tISR-2, particularly suppression of the increased hepatic phosphorylation of both Ser^307^ in IRS-1 and Ser^731^ in IRS-2, further confirms improvement of hepatic insulin signaling by rosiglitazone. Thus, these results suggest that rosiglitazone improves hepatic insulin resistance, which may contribute to the amelioration of systemic insulin resistance in OLETF rats. Up to now, no evidence has been found to demonstrate that rosiglitazone directly affects hepatic total or phosphorylated IRS-1 and IRS-2 expression. It has been demonstrated that treatment of the L-02 cells derived from adult human liver with rosiglitazone does not affect glucose consumption and fails to affect mRNA expression of IRS-2 [32]. Thus, rosiglitazone-elicited suppression of hepatic phosphorylated IRS-1 and IRS-2 protein expression might be caused by the regulation of the upstream factors.

Inflammation, unusual lipid metabolism, and insulin resistance are interlinked components of the metabolic syndrome. Excessive hepatic lipid accumulation is closely associated with hepatic insulin resistance [33]. Inflammation is the basis of the metabolic disorders of insulin resistance and type 2 diabetes; chronic inflammation plays a pivotal role in the development of insulin resistance in obesity [34–37]. NF-*κ*B is a transcription factor and primarily regulates inflammatory responses; thus, NF-*κ*B plays a critical role in inflammation [38]. IKK-*β* is necessary for activation of NF-*κ*B during chronic inflammation [39]. It has been demonstrated that activation of IKK-*β* and NF-*κ*B results in local and systemic insulin resistance in rats [40]. In contrast, disruption of IKK-*β* in hepatocytes protects mice from insulin resistance in response to a high-fat diet, obesity, or ageing, suggesting the link of inflammation to obesity-induced insulin resistance by IKK-*β* in the liver [41]. Targeted disruption of IKK-*β* has also been found to reverse genetically occurred obesity and high-fat-diet-induced insulin resistance in mice [42]. Thus, inflammation and the IKK-*β*/I*κ*B/NF-*κ*B pathway actually form an axis in obesity- and diet-induced insulin resistance [43]. It has been demonstrated that rosiglitazone treatment improves high-fat- and high-sucrose-induced hepatic steatosis and inflammation in rats [44], suppresses skeletal muscle inflammation by blocking the NF-*κ*B pathway in OLETF rats [45], and inhibits chronic pancreatitis-induced activation of the hepatic IKK-*β*/NF-*κ*B pathway in rats [6]. In the present study, OLETF rats showed excessive hepatic triglyceride accumulation. Furthermore, the plasma ALT activity was also higher than that of LETO rats, indicating liver injury due to chronic inflammation. Treatment with rosiglitazone attenuated fatty liver and inhibited the increase in plasma ALT activity. More importantly, the upregulated hepatic expression of both phosphorylated IKK-*α*/*β* and NF-*κ*Bp65 proteins in OLETF rats was suppressed. Thus, it is suggested that inhibition of hepatic IKK-*β*/I*κ*B/NF-*κ*B-mediated chronic inflammation and attenuation of fatty liver are responsible for rosiglitazone-elicited improvement of hepatic insulin resistance in OLETF rats.

Adiponectin is mostly secreted by adipose tissue and has a positive impact on insulin sensitivity [16]. Under obese situation, the synthesis and release of adiponectin from adipose tissue are decreased, and the low serum adiponectin concentration resulting from obesity is associated with insulin resistance; in contrast, an increase in adiponectin by various interventions decreases systemic insulin resistance [16]. Serum adiponectin concentration has been found to be negatively associated with the severity of hepatic steatosis, inflammation, and fibrosis [16]. Treatment with TDZs has been demonstrated to improve hypoadiponectinemia, hepatic steatosis, lobular inflammation, and insulin resistance in patients with nonalcoholic steatohepatitis [46]. In the liver, ADNRs mediate the insulin-sensitizing effect of adiponectin through inhibiting NF-*κ*B activation to suppress inflammation and through activating both AMPK and PPAR-*α* pathways to decrease gluconeogenesis, de novo lipogenesis, and free fatty acid influx and to increase fatty acid *β*-oxidation [17]. It was found that rosiglitazone at higher concentrations (100–5000 nmol/L) directly upregulated ADNR-2 protein expression but did not affect ADNR-1 protein level in HepG2 cells [47]. In the present study, oral administration of rosiglitazone at low dosage (3 mg/kg) restored adipose adiponectin expression and normalized the decreased plasma adiponectin concentration in OLETF rats. Furthermore, rosiglitazone did not only enhance the hepatic protein expression of ADNR-2 but also increased the hepatic ADNR-1 protein level. Thus, these results imply that rosiglitazone-treatment-elicited insulin-sensitizing action in the liver is mediated by upregulation of hepatic ADNRs through increasing the secretion and release of adiponectin in adipose tissue.

## 5. Conclusion

**Figure 8:**
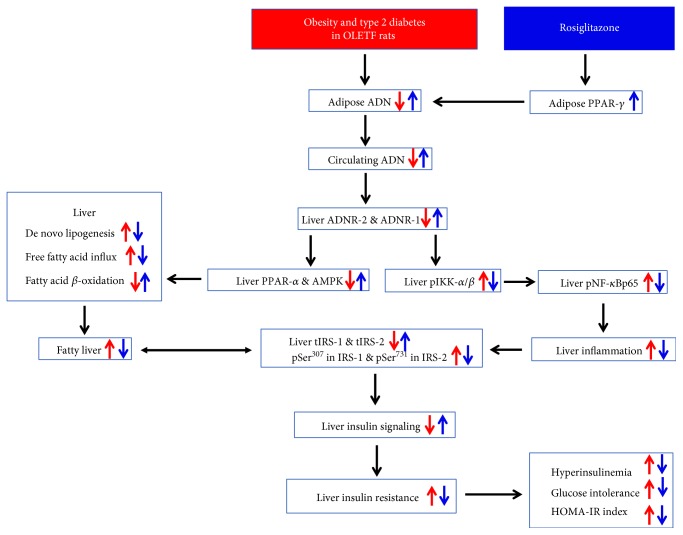
The proposed mechanisms underlying the rosiglitazone-elicited insulin-sensitizing action at the adipose tissue-liver axis.

Taken together, the present results demonstrate that rosiglitazone elicits an adiponectin-mediated insulin-sensitizing action at the adipose tissue-liver axis in genetically occurred obese rats (the proposed underlying mechanisms of action are shown in Figure 8). Our findings may provide better understanding of the mechanisms underlying the insulin-sensitizing action of the TZD.

## Data Availability

The data used to support the findings of this study are available from the corresponding author upon request.

## Abbreviations

ADN: Adiponectin
ADNR: Adiponectin receptor
ALT: Alanine aminotransferase
AMPK: 5-AMP-activated protein kinase
AUC: Area under the curve
HOMA-IR: The homeostasis model assessment of insulin resistance
IKK: Inhibitory-*κ*B kinase
pIKK: Phosphorylated inhibitory-*κ*B kinase protein
NF: Nuclear factor
pNF: Phosphorylated nuclear factor
IRS: Insulin receptor substrate
tIRS: Total insulin receptor substrate
LETO: Long-Evans Tokushima Otsuka
NEFA: Nonesterified fatty acids
OGTT: Oral glucose tolerance test
OLETF: Otsuka Long-Evans Tokushima Fatty
PPAR: Peroxisome proliferator-activated receptor
TZD: Thiazolidinedione
WAT: White adipose tissue.
